# Hypoglycaemia Metabolic Gene Panel Testing

**DOI:** 10.3389/fendo.2022.826167

**Published:** 2022-03-29

**Authors:** Arianna Maiorana, Francesca Romana Lepri, Antonio Novelli, Carlo Dionisi-Vici

**Affiliations:** ^1^Division of Metabolism, Department of Pediatrics Subspecialties, Ospedale Pediatrico Bambino Gesù, IRCCS (Istituto di Ricovero e Cura a Carattere Scientifico), Rome, Italy; ^2^Laboratory of Medical Genetics, Translational Cytogenomics Research Unity, Ospedale Pediatrico Bambino Gesù, IRCCS (Istituto di Ricovero e Cura a Carattere Scientifico), Rome, Italy

**Keywords:** hypoglycemia, next generation sequencing, NGS, whole exome sequencing, whole genome sequencing

## Abstract

A large number of inborn errors of metabolism present with hypoglycemia. Impairment of glucose homeostasis may arise from different biochemical pathways involving insulin secretion, fatty acid oxidation, ketone bodies formation and degradation, glycogen metabolism, fructose and galactose metabolism, branched chain aminoacids and tyrosine metabolism, mitochondrial function and glycosylation proteins mechanisms. Historically, genetic analysis consisted of highly detailed molecular testing of nominated single genes. However, more recently, the genetic heterogeneity of these conditions imposed to perform extensive molecular testing within a useful timeframe *via* new generation sequencing technology. Indeed, the establishment of a rapid diagnosis drives specific nutritional and medical therapies. The biochemical and clinical phenotypes are critical to guide the molecular analysis toward those clusters of genes involved in specific pathways, and address data interpretation regarding the finding of possible disease-causing variants at first reported as variants of uncertain significance in known genes or the discovery of new disease genes. Also, the trio’s analysis allows genetic counseling for recurrence risk in further pregnancies. Besides, this approach is allowing to expand the phenotypic characterization of a disease when pathogenic variants give raise to unexpected clinical pictures. Multidisciplinary input and collaboration are increasingly key for addressing the analysis and interpreting the significance of the genetic results, allowing rapidly their translation from bench to bedside.

## 1 Introduction

Hypoglycemia is associated with a large number of inborn errors of metabolism (IEM). The alteration of biochemical pathways involving carbohydrate, protein and lipid metabolism often leads to an impairment of glucose homeostasis (1–3). Although biochemical features of hypoglycemia are useful tools to undercover the underlying pathology, overlapping or unspecific features make arduous to reach a diagnosis at a short time. Indeed, metabolic diseases which can present with intermittent or persistent hypoglycemia include disorders of carbohydrate metabolism (glycogen storage diseases [GSDs], gluconeogenesis defects, hereditary fructose intolerance [HFI], galactosemia), hyperinsulinemic hypoglycemia (HI), fatty acid oxidation defects (FAODs), ketogenesis and ketolysis defects, mitochondrial DNA depletion syndromes, and some aminoacidopathies (maple syrup urine disease, hepato-renal tyrosinemia [HT1], adenosine kinase deficiency). The establishment of a precise diagnosis is crucial to start specific nutritional and pharmacological therapies. Due to the multitude of genes associated with IEM, standard molecular approaches with Sanger sequencing for single genes would result expensive and time-consuming. In the last decade, next-generation sequencing (NGS) technologies have become essential tool for their rapid turnaround time and coverage in the field of metabolic diseases.

## 2 Clinical and Biochemical Characterization of Hypoglycemia

According to current recommendations, hypoglycemia is defined as spontaneous symptomatic hypoglycemia and/or plasma glucose concentration <3.3 mmol/L (<60 mg/dL), or < 2.8 mmol/L (<50 mg/dL) after a provocative fasting test. In the differential diagnosis of hypoglycemia, it is important to evaluate the timing of hypoglycemia in relation to fasting state, eventual associated signs of visceral (hepatomegaly/hepatopathy, myopathy, cardiomyopathy) and neurologic involvement, and suggestive signs of hypopituitarism or adrenal insufficiency. Furthermore, a specific laboratory work-up needs to be performed at the time of hypoglycemia. Laboratory assays include routine analyses (plasma glucose, blood gas analysis, lactate, ammonia, uric acid, liver and muscle enzymes, free or non-esterified fatty acids [NEFA], triglycerides, blood and urinary ketones), endocrine (insulin, C-peptide, growth hormone, cortisol) and metabolic investigations (blood acylcarnitines, plasma aminoacids, urinary organic acids and transferrin isoforms) (1, 3).

The characterization in non-ketotic (or hypoketotic) and ketotic hypoglycemia (KH) distinguishes two major categories of disease: the first includes HI, hypopituitarism, GSD type I, FAOD and ketogenesis defects, the latter covers idiopatic ketotic hypoglycemia (IKH), single hormonal defects (cortisol deficiency, GH deficiency) and all the others metabolic diseases. Within non-ketotic hypoglycemias, suppressed NEFA are typical in HI and hypopituitarism.

## 3 IEM presenting With Hypoglycemia in Childhood

### 3.1 Disorders of Carbohydrate Metabolism

#### 3.1.1 Hyperinsulinemic Hypoglycemia

The diagnosis of HI is defined by detectable plasma insulin level (>2–3 μU/ml) at the time of hypoglycemia or by signs of inappropriate excess of insulin, such as suppressed NEFA (<1.7 mM), hypoketonemia (<1.8 mM), a hyperglycemic response to i.m. glucagon (delta glucose>30 mg/dl in 30 min) and a high glucose demand (>10 mg/kg/min in neonates) (4–6).

Mutations in *ABCC8* and *KCNJ11* (encoding for the SUR1 and Kir6.2 subunits of the K_ATP_ channel, respectively) account for more than half of HI cases (7–11) and have been associated with two histological aspects of the endocrine pancreas. A diffuse form extended to all pancreatic β-cells, inherited as either autosomal recessive or dominant trait, and a focal form, which results from the combination of a paternally inherited germinal mutation and a somatic loss of heterozygosity of the maternal allele in a restricted group of β-cells (12). The two forms have different management and outcome, and hence differential diagnosis is a crucial point. Indeed, partial pancreatectomy is the elective procedure for focal HI and allows the complete recovery from hypoglycemia. Therefore, finding a genotype suggestive for a focal form determines the subsequent diagnostic pathway through 18F-DOPA PET/CT (13).

Beyond *ABCC8* and *KCNJ11*, mutations in pancreatic genes involved in fatty acid oxidation, energy and aminoacid metabolism and transcription factors give rise to diffuse forms of HI: *GCK*, *GLUD1*, *HADH*, *HNA4a*, *HNF1a*, *SLC16A1*, *UCP2*, *HK1*, *INSR* (13, 14). Except GLUD1-HI, in which hyperammonemia is a characteristic finding, in all other diffuse forms there is no recognized biomarker, and molecular analysis is the only tool to make a specific diagnosis.

Diffuse HI is treated with medical therapy (diazoxide, octreotide), and only in case of unresponsiveness, a near-total pancreatectomy is required. However, this procedure is often associated with increased risk of diabetes and exocrine pancreatic failure (15–18), and does not guarantee the remission of hypoglycemia (17). For these reasons, in severe unresponsive forms of GCK-HI, a therapeutic approach with ketogenic diet was recently successfully proposed as elective treatment, because patients recovered from epilepsy, intellectual disabilities and symptoms of recurrent hypoglycemia (19).

Furthermore, specific mutations p.R63W and LRG_483t1:c.427-1G>A in *HNF4α* cause HI associated to hepatomegaly and renal Fanconi syndrome, a phenotype similar to Fanconi-Bickel syndrome (due to inactivating mutations of *SLC2A2* resulting in nonfunctional glucose transporter 2, GLUT2). This *HNF4α* mutations might decrease the expression of *SLC2A2* in both liver and kidney, and are responsive to diazoxide therapy, unlike the Fanconi-Bickel syndrome (20, 21).

HI also occurs in some congenital disorders of glycosylation, such as PMM2-CDG (22), PMI-CDG (23), ALG6-CDG (24), ALG3-CDG (25), and PGM1-CDG (26, 27).

PMI-CDG and PGM1-CDG are treatable disorders with mannose and galactose therapy, respectively. Therefore, the genetic characterization leads the therapeutic choices.

Several genetic syndromes have been particularly associated with HI or with KH. Syndromic hypoglycemias caused by mutations in known genes are listed in **Table 1**. However, syndromic conditions due to chromosomal aberrations (e.g. Turner syndrome, Trisomy 13, Trisomy 21) or genetic deletions (e.g. Usher syndrome caused by contiguous gene deletion including *USH1C* and *ABCC8*; 16p11.2 deletion, 22q11.2 deletion, 9p deletion) or epigenetic alterations (e.g. Beckwith-Wiedemann, Silver-Russel, Prader Willi syndromes) or undiagnosed dysmorfisms have been reported to be associated with hypoglycemia (13, 28).

**Table 1:** Genes associated to Hypoglycemia.

GENE	Disease	Inheritance	OMIM
***Hyperinsulinemic Hypoglycemia***			
*ABCC8*	Hyperinsulinemic hypoglycemia, familial, 1	AD, AR	# 256450
*KCNJ11*	Hyperinsulinemic hypoglycemia, familial, 2	AD, AR	# 601820
*GLUD1*	Hyperinsulinism-hyperammonemia syndrome	AD	# 606762
*GCK*	Hyperinsulinemic hypoglycemia, familial, 3	AD	# 602485
*HADHSC*	Hyperinsulinemic hypoglycemia, familial, 4	AR	# 609975
*HNF4A*	MODY, type I	AD	# 125850
*SLC16A1*	Hyperinsulinemic hypoglycemia, familial, 7	AD	# 610021
*UCP2*	Obesity, susceptibility to, BMIQ4	AD	# 601693
*HNF1A*	MODY, type III	AD	# 600496
*INSR*	Hyperinsulinemic hypoglycemia, familial, 5	AD	# 609968
*HK1*	Hexokinase 1	AR	*142600
***Glycogen storage diseases***			
*G6PC1*	Glycogen storage disease Ia	AR	# 232200
*SLC37A4*	Glycogen storage disease Ib	AR	# 232220
*AGL*	Glycogen storage disease III	AR	# 232400
*GBE1*	Glycogen storage disease IV	AR	# 232500
*PYGL*	Glycogen storage disease VI	AR	# 232700
*PHKA2*	Glycogen storage disease IXa	XLR	# 306000
*PHKB*	Glycogen storage disease IXb	AR	# 261750
*PHKG2*	Glycogen storage disease IXc	AR	# 613027
*SLC2A2*	Fanconi-Bickel syndrome	AR	# 227810
*GYS2*	Glycogen storage disease 0, liver	AR	# 240600
***Gluconeogenesis defects***			
*FBP1*	Fructose-1,6-bisphosphatase deficiency	AR	# 229700
*PCK1*	Phosphoenolpyruvate carboxykinase deficiency	AR	# 261680
***Hereditary fructose intolerance***			
*ALDOB*	Hereditary fructose intolerance	AR	# 229600
***Galactosemia***			
*GALT*	Galactosemia 1	AR	# 230400
*GALE*	Galactose epimerase deficiency	AR	# 230350
***Congenital disorders of glycosylation***			
*PMM2*	Congenital disorder of glycosylation, type Ia	AR	# 212065
*MPI*	Congenital disorder of glycosylation, type Ib	AR	# 602579
*ALG6*	Congenital disorder of glycosylation, type Ic	AR	# 603147
*ALG3*	Congenital disorder of glycosylation, type Id	AR	# 601110
*PGM1*	Congenital disorder of glycosylation, type It	AR	# 614921
***β-oxidation defects***			
*SLC22A5*	Carnitine deficiency, systemic primary	AR	# 212140
*CPT1A*	CPT deficiency, hepatic, type IA	AR	# 255120
*SLC25A20*	Carnitine-acylcarnitine translocase deficiency	AR	# 212138
*CPT2*	CPT II deficiency	AR	# 600649
*ACADVL*	VLCAD deficiency	AR	# 201475
*HADHA*	LCHAD deficiency	AR	# 609016
*HADHB*	Trifunctional protein deficiency	AR	# 609015
*ACADM*	Acyl-CoA dehydrogenase, medium chain, deficiency	AR	# 201450
*ACADS*	Acyl-CoA dehydrogenase, short-chain, deficiency	AR	# 201470
*HADH*	3-hydroxyacyl-CoA dehydrogenase deficiency	AR	# 231530
*ETFA*	Glutaric acidemia IIA	AR	# 231680
*ETFB*	Glutaric acidemia IIB	AR	# 231680
*ETFDH*	Glutaric acidemia IIC	AR	# 231680
***Ketogenesis defects***			
*HMGCS2*	HMG-CoA synthase-2 deficiency	AR	# 605911
*HMGCL*	HMG-CoA lyase deficiency	AR	# 246450
***Ketolysis defects***			
*ACAT1*	Beta-ketothiolase deficiency	AR	# 203750
*SLC16A1*	Monocarboxylate transporter 1 deficiency	AD, AR	# 616095
***Organic acidemia***			
*MMUT*	Methylmalonic aciduria, mut (0) type	AR	# 251000
*MMAA*	Methylmalonic acidemia, cblA	AR	# 251100
*MMAB*	Methylmalonic acidemia, cblB	AR	# 251110
*PCCA*	Propionic acidemia	AR	# 606054
*PCCB*	Propionic acidemia	AR	# 606054
*IVD*	Isovaleric acidemia	AR	# 243500
***Maple syrup urine disease***			
*BCKDHA*	Maple syrup urine disease, type Ia	AR	# 248600
*BCKDHB*	Maple syrup urine disease, type Ib	AR	# 248600
*DBT*	Maple syrup urine disease, type II	AR	# 248600
*DLD*	Maple syrup urine disease, type III	AR	# 246900
***Tyrosinemia type 1***			
*FAH*	Hepato-renal tyrosinemia (Tyrosinemia, type I)	AR	# 276700
***Adenosine kinase deficiency***			
*ADK*	Adenosine kinase deficiency	AR	# 614300
***Mitochondrial DNA depletion syndrome***			
*MPV17*	Mitochondrial DNA depletion syndrome 6 (hepatocerebral type)	AR	# 256810
*DGUOK*	Mitochondrial DNA depletion syndrome 3 (hepatocerebral type)	AR	# 251880
*SUCLG1*	Mitochondrial DNA depletion syndrome 9	AR	# 245400
*TWNK*	Mitochondrial DNA depletion syndrome 7 (hepatocerebral type)	AR	# 271245
*TFAM*	Mitochondrial DNA depletion syndrome 15 (hepatocerebral type)	AR	# 617156
*POLG*	Mitochondrial DNA depletion syndrome 4A	AR	# 203700
***Other genes***			
*APPL1*	Maturity-onset diabetes of the young, type 14	AD	# 616511
*BLK*	Maturity-onset diabetes of the young, type 11	AD	# 613375
*CEL*	Maturity-onset diabetes of the young, type VIII	AD	# 609812
*GK*	Glycerol kinase deficiency	XLR	# 307030
*HNF1B*	Type 2 diabetes mellitus	AD	# 125853
*IGF1R*	Insulin-like growth factor I, resistance to	AD, AR	# 270450
*INS*	Hyperproinsulinemia	AD	# 616214
*KLF11*	Maturity-onset diabetes of the young, type VII	AD	# 610508
*MAFA*	Insulinomatosis and diabetes mellitus	AD	# 147630
*NEUROD1*	Diabetes mellitus, type 2 susceptibility to	AD	# 125853
*PAX4*	Diabetes mellitus, type 2 susceptibility to	AD	# 125853
*PDX1*	Diabetes mellitus, type 2 susceptibility to	AD	# 125853
*SLC5A1*	Glucose/galactose malabsorption	AR	# 182380
*SLC25A13*	Citrullinemia type II	AR	# 603859
*ACAD9*	ACAD9 deficiency	AR	# 611103
***Syndromic Hypoglycemia***			
*CDKN1C*	Beckwith-Wiedemann syndrome	AR	# 130650
*CACNA1C*	Timothy syndrome	AD	# 601005
*NSD1*	Sotos syndrome 1	AD	# 117550
*GPC3*	Simpson-Golabi-Behmel syndrome	XLR	# 312870
*HRAS*	Costello syndrome	AD	# 218040
*DIS3L2*	Perlman syndrome	AR	# 267000
*KMT2D*	Kabuki syndrome 1	AD	# 147920
*KDM6A*	Kabuki syndrome 2	XLD	# 300867
*GHR*	Laron syndrome	AR	# 262500
*PHOX2B*	Ondine (central hypoventilation syndrome)	AD	# 209880
*TRMT10A*	Microcephaly, short stature, and impaired glucose metabolism 1	AR	# 616033
*ARID1B*	Coffin-Siris syndrome 1	AD	# 135900
*CHD7*	CHARGE syndrome	AD	# 214800
*CREBBP*	Rubinstein-Taybi syndrome 1	AD	# 180849
*EP300*	Rubinstein-Taybi syndrome 2	AD	# 613684
*JAG1*	Alagille syndrome	AD	# 118450
*RNF125*	Tenorio syndrome	AD	# 610432
*AKT2*	Hypoinsulinemic hypoglycemia with hemihypertrophy	AD	# 240900
*PIK3R2*	Megalencephaly-polymicrogyria syndrome	AD	# 603387
*AKT3*	Megalencephaly-polymicrogyria syndrome	AD	# 615937
*PIK3CA*	Phosphatidylinositol 3-kinase, catalytic, alpha		*171834
*CCND2*	Cyclin D2		*123833
*APC2*	Cortical dysplasia, complex, with other brain malformations	AR	# 618677
*PLAGL1*	Silver-Russell syndrome 4	AD	# 618907
*CACNA1D*	Primary aldosteronism, seizures, and neurologic abnormalities	AD	# 615474
***New Genes***			
*NCOR1*	Nuclear Receptor corepressor 1		* 600849
*IGF2BP1*	Insulin like growth factor 2 mRNA binding protein 1		* 608288
*SLC5A2*	Renal glucosuria	AD, AR	# 233100
*NEK11*	NIMA related kinase 2		* 604043
*FOXA2*	Hepatocyte nuclear factor 3, beta		* 600288
*EIF2S3*	MEHMO syndrome	XLR	# 300148
*DNAJC3*	Neuroendocrine developmental disorder with insulin dysregulation	AR	# 616192

#OMIM phenotype description, molecular basis known.

*OMIM gene description.

#### 3.1.2 Glycogen Storage Diseases

GSDs are IEM involving synthesis and degradation of glycogen, resulting in a failure to convert glycogen into energy, and in a glycogen accumulation in multiple organs. Glycogen is a branched polymer of glucose molecules. After a meal, plasma glucose increases and stimulates the storage of excess glucose in form of cytoplasmic glycogen in many tissues as liver, skeletal muscle, heart and kidney. Hepatic GSDs present with hypoglycemia, particularly the major types GSD Ia, Ib and III. Dietary treatment is the cornerstone of management aiming at maintenance of euglycaemia, prevention of secondary metabolic perturbations, and long-term complications affecting multiple organs, such as liver (hepatocellular adenomas and carcinomas), heart (cardiomyopathy), muscle (myopathy), kidneys (proteinuria, renal insufficiency, stones), and bone (osteopenia, osteoporosis). According to GSD type and age, patients are treated with hyperglucidic diet with frequent feeds, continuous nocturnal gastric drip feeding or late evening meal supplemented with uncooked cornstarch, or restriction of mono- and disaccharides, or hyperproteic diet (29).

GSD type I is a rare disease of variable clinical severity that primarily affects the liver and kidney. It is caused by deficient activity of the glucose 6-phosphatase enzyme (GSD Ia) or a deficiency in the microsomal transport proteins for glucose 6-phosphate (GSD Ib), resulting in excessive accumulation of glycogen and fat in the liver, kidney, and intestinal mucosa. Patients have a wide spectrum of clinical and biochemical manifestations, including hepatomegaly, growth retardation, hypoketotic hypoglycemia, hyperlactatemia, metabolic acidosis, hyperuricemia and hyperlipidemia. Since both glycogenolysis and gluconeogenesis are affected, individuals with GSD type Ia typically manifest hypoglycemia in infancy when the interval between feedings is extended to 2-3 hours. Rate of complications and disease severity are variable. In addition, patients with GSD type Ib manifest neutropenia and neutrophil dysfunction, recurrent infections and inflammatory bowel disease (30). In the first two years of life, the phenotype of the two forms is undistinguishable until neutropenia appears. Patients are treated with frequent feeds of hyperglucidic, hypolipidic diet, added with maltodextrin and cornstarch, and with nocturnal enteral feeding in the first year of life. Recently, in GSD type Ib a novel treatment with empagliflozin appeared effective in controlling neutrophil dysfunction and inflammatory bowel disease (31).

GSD type III is caused by recessive mutations in the *AGL* gene with consequent deficiency of the glycogen debranching enzyme. Patients manifest hepatomegaly, growth retardation, KH, hyperlipidemia and elevated liver enzymes. Phenotypically, patients can be further classified into having GSD type IIIa (85%), with involvement of the liver, heart, and skeletal muscle, or GSDIIIb (15%), in which only the liver is affected (32). Since gluconeogenesis is unaffected, patients with GSD type IIIb are commonly treated with a high protein diet, and cornstarch if necessary. GSD type IIIa with cardiomyopathy is an elective indication for ketogenic diet, which completely reverses the cardiac hypertrophy (33–36).

The other forms of hepatic GSDs type IV, VI, IXa, IXb, IXc typically present with hepatomegaly, elevated liver enzymes, dyslipidemia, growth retardation, renal tubular dysfunction and can present with KH. Liver GSDs take overlapping symptoms and can be clinically indistinguishable.

GSD type IV is caused by recessive mutations in the *GBE1* gene, which leads to 1,4-α-glucan-branching enzyme deficiency. GBE deficiency involves the liver, the neuromuscular system and the heart. In the classical (progressive) hepatic phenotype, infants develop hepatomegaly, hypoglycemia, hypotonia, and developmental delay during the first months of life, with rapid progression to portal hypertension, ascites and liver cirrhosis between the second and fourth year of life. A nonprogressing form with exclusively liver involvement has been reported in a few cases. Neuromuscular symptoms may appear from fetal to adult age. The most severe form starts *in utero* with decreased fetal movements, arthrogryposis, hypoplastic lungs, and may cause perinatal death. Patients are treated with hyperglucidic diet plus cornstarch, nocturnal enteral feeding, protein enrichment, and in the more severe form, with liver transplantation (37).

GSD type VI presents as a relatively mild disorder in infancy and childhood (38–40). It is caused by recessive mutations or deletions of the *PYGL* gene (deficiency of liver phosphorylase) (41).

GSD type IX is the most frequent hepatic GSD resulting from a deficient liver phosphorylase kinase (PhK) system. GSD type IXa (*PHKA2* mutations) is the most common subtype of liver PhK deficiency, accounting for 75% of GSD type IX, with an X-linked inheritance. Patients usually manifest hepatomegaly, hepatopathy, hypoglycemia and renal tubulopathy with a milder or benign courses. Conversely, patients with GSD type IXc (*PHKG2* mutations) have more severe clinical features such as mild gross motor delays, hypoglycemia, liver fibrosis and cirrhosis in childhood (42). Patients are treated with high protein diet, and cornstarch if needed.

Fanconi-Bickel syndrome (also known as GSD type XI) is caused by mutations in the GLUT2 (*SLC2A2*) gene. It is characterized by glycogen accumulation in liver and kidneys, with fasting hypoglycemia, hepatomegaly, tubular nephropathy (glucosuria, proteinuria, phosphaturia, bicarbonate wasting, and aminoaciduria), rickets, failure to thrive and short stature. The phenotypic variability ranges from mild presentation to diabetes mellitus (43, 44). Patients are treated with hyperglucidic diet with low content of galactose.

In the hepatic GSD type 0, caused by mutations in *GYS2*, glycogen synthesis is impaired. As glucose cannot be converted to glycogen, patients manifest fasting hypoglycemia and postprandial hyperglycemia. Postprandial hyperlactatemia also develops for the conversion of meal-derived carbohydrates to lactate. Fasting ketotic hypoglycemia usually manifests in late infancy when overnight feedings are stopped. Since gluconeogenesis and fatty acid oxidation are unaffected, in general the cognitive function is normal. Short stature and osteopenia are common features (45–48). The disease is underdiagnosed. Patients are treated with frequent feeds of hyperglucidic and hyperproteic diet.

#### 3.1.3 Gluconeogenesis Defects

Gluconeogenesis plays an essential role in glucose homeostasis. Through this pathway, amino acids, lactate, glycerol, and other non-carbohydrate substrates are converted into glucose to meet energy demands under prolonged starvation, infections or metabolic stress (49).

Fructose-1,6-phosphatase (*FBP1*) deficiency manifests in the neonatal period or later on with KH, iperlactatemia, metabolic acidosis, hyperuricemia, hepatomegaly during decompensations. Patients present hyperalaninemia and glucagon-unresponsiveness. Elevated levels of glycerol 3-phosphate can be found in urine organic acid analysis (50). Alterations of consciousness can progress into coma. Episodes are typically triggered by fasting, infections, or ingestion of large amounts of fructose. Patients need to avoid fasting, they are treated with frequent feeds, often with added cornstarch. Tolerance to fasting improves with age (51). The presence of urinary glycerol 3-phosphate puts the disease in differential diagnosis with the Glycerol kinase deficiency, an X-linked recessive disorder characterized by hyperglycerolaemia and glyceroluria. Indeed, children affected by the juvenile form of the latter condition may present with Reye-like episodes of vomiting, metabolic acidosis and KH with progressive unconsciousness during intercurrent illnesses, and “pseudohypertriglyceridaemia” as a result of a raised plasma glycerol (52).

Cytosolic Phosphoenolpyruvate carboxykinase (*PEPCK, PCK1*) deficiency begins in neonatal age or after a few months. Beyond the phenotypic alterations described for FBP1 deficiency, patients display mostly progressive neurologic involvement with hypotonia, developmental delay, epilepsy, spasticity, microcephaly and multiorgan damage with hepatomegaly, hepatocellular dysfunction, cardiomyopathy, muscular weakness, renal tubular acidosis, and failure to thrive. The clinical picture may also mimic Reye syndrome (53, 54). Urine organic acids profile shows low or absent ketonuria with presence of fumarate, adipate, succinate, 2-ketoglutarate and glutarate, sometimes ethylmalonate, and in some patients a profile similar to those seen in defects of ketogenesis has been reported (55). Treatment is based on high carbohydrate diet plus cornstarch, and avoidance of fasting.

#### 3.1.4 Hereditary Fructose Intolerance

Hereditary fructose intolerance is an autosomal-recessive disorder caused by deficiency of aldolase B. Upon introduction of fructose-containing foods patients manifest abdominal pain, nausea, recurrent vomiting, hypoglycemia, lactic acidemia, hypophosphatemia, hyperuricemia in case of acute fructose intoxication. Parenteral intravenously administration of fructose, sorbitol, or sucrose may be life threatening for severe hypoglycemia and acute hepato-renal failure, and must be rigorously avoided (56). ATP depletion with toxic effect on glycogenolysis and gluconeogenesis is responsible of hypoglycemia. Many individuals with HFI exhibit a self-imposed aversion to fructose-containing foods, sufficiently to prevent an acute intoxication. However, prolonged fructose intake leads to poor feeding, vomiting, failure to thrive, hepatomegaly, liver and renal tubular dysfunction that might lead to irreversible liver and kidney damage (57, 58). Upon dietary restriction of fructose, symptoms resolve and normal growth and development is achieved. Therefore, individuals with HFI need to be treated with a fructose, sorbitol, sucrose-restricted diet. The disease can be misdiagnosed because some individuals can only manifest fruit aversion. However, the ingestion of certain medicinal formulations containing fructose or analogue sugars can cause severe hypoglycemia and acute hepato-renal failure (58).

#### 3.1.5 Galactosemia

Infants with galactosemia typically present within the first few days of life with liver failure with coagulopathy, jaundice, hepatomegaly, hypoglycemia, seizures, cerebral edema after exposure to dietary galactose in the form of breastmilk or standard infant formulas. Additional findings may include poor weight gain, lethargia, renal failure, cataracts, vitreous hemorrhage and Escherichia coli sepsis. The disease with presentation of acute liver failure and hypoglycemia is caused by recessive mutations of galactose-1-phosphate uridyltransferase (*GALT*) and uridine diphosphate-galactose 4-epimerase (*GALE*). Long-term outcomes are oro-motor dyspraxia, intellectual disabilities, tremors and ataxia, ovarian dysfunction, osteoporosis. The therapy consists in low galactose/lactose diet (59).

#### 3.1.6 Congenital Disorders of Glycosylation

Congenital disorders of glycosylation (CDGs) are complex rare diseases involved in protein glycosylation with functional consequences in protein folding, endocrine function, immune response, coagulation, cell interaction and signal transduction. A characteristic marker is altered glycosylation of transferrin visible at isoelectrofocusing of serum transferrin.

Phosphomannomutase 2 (PMM2)-CDG, Glucosyltransferase 1 (ALG6)-CDG and Mannosyltransferase 6 (ALG3)-CDG are complex disorders with multiorgan involvement and can present with HI.

PMM2-CDG is characterized by a neurological picture of internal strabismus, psychomotor disability, ataxia, cerebellar hypoplasia, epilepsy and classical features of inverted nipples and abnormal subcutaneous adipose tissue distribution. Nearly all other organs can be involved (22). ALG6-CDG presents with psychomotor disability, neurological symptoms, behavioural problems, skeletal abnormalities, and often protein-losing enteropathy (24). ALG3-CDG leads to severe neurological and skeleton involvement (25).

Phosphomannoisomerase (PMI)-CGD is a complex non neurologic syndrome characterized by protein-losing enteropathy, hepatopathy/liver failure, coagulopathy, HI and normal development. PMI catalyzes the conversion of fructose-6-P in mannose-6-P. HI patients are responsive to diazoxide. Therapy with mannose (which can be converted to mannose-6-P by hesokinase enzyme) restores intestinal and hepatic function, coagulation, hypoglycemia and the isoelectrofocusing of serum transferrin (23).

Phosphoglucomutase 1 (PGM1)-CDG has a complex phenotype characterized by hepatopathy, myopathy, exercise-induced rhabdomyolysis, cardiomyopathy, bifid uvula, growth retardation, coagulation and endocrine alterations. It is also called GSD type XIV, because patients show a combination of fasting KH, with post-prandial HI. Since PGM1 catalyzes the transfer of phosphate between glucose-1-P and glucose-6-P, the proposed mechanisms are impaired carbohydrate metabolism of the glycogen pathway for fasting KH, and a lower glucose threshold for insulin secretion caused by the increased glucose-6-P for post-prandial HI. Therapy with oral galactose improves hypoglycemia, endocrine abnormalities and coagulation as well as transferrin glycosylation pattern (26, 27).

### 3.2 Disorders of Lipid Metabolism

#### 3.2.1 β-Oxidation Defects

The oxidation of fatty acids in mithocondria plays an important role in energy production. During late stages of fasting, fatty acids are released from adipose tissue triglyceride stores. Their oxidation spares glucose consumption and the need to use proteins to form glucose. Furthermore, the oxidation of fatty acids by the liver provides energy for gluconeogenesis and ureagenesis. Long-chain fatty acids are used by the heart and skeletal muscle during sustained exercise. In the liver, they are converted in ketone bodies, which serve as a fuel for brain, and thus further reduce the need for glucose utilization. Therefore, FAODs are characterized by fasting or stress induced hypoketotic hypoglycemia with increased NEFA, and presents with three major signs of hepatic, skeletal muscle and cardiac involvement: raised liver and/or muscle enzymes (hepatopathy/rabdomyolysis) with or without hypoglycemia, cardiomyopathy and arrhythmias. Specific biomarkers are abnormal acylcarnitines and/or urinary organic acids. The foundation of therapy is to prevent metabolic decompensations, avoiding fasting stress.

Defects in carnitine cycle, in very long chain-, long chain-, medium chain-, short chain dehydrogenases and in the electron transfer pathway cause different forms of FAODs, which require different nutritional (low-fat diet and medium chain triglycerides administration in some long chain FAODs) and therapeutic approaches or no approach. In addition, a single defect may have a variety of clinical manifestations even within the same family, as the case of multiple acyl-CoA dehydrogenase deficiencies (MADD), which ranges from hypoketotic hypoglycaemia, metabolic acidosis, cardiomyopathy to leukodystrophy, neurodevelopmental delay and myopathy (60). Genetic analysis has a pivotal role for diagnosis and prognosis establishment, and allows to personalize the treatment.

#### 3.2.2 Ketogenesis Defects

During fasting, ketone bodies are important fuels for many tissues, such as brain, heart and skeletal muscle. Disorders of ketone bodies formation present either in the first few days of life or later in childhood, during infections, prolonged fasting or other metabolic stress. During decompensation patients present encephalopathy with vomiting and a reduced level of consciousness, and often hepatomegaly. The biochemical features, hypoketotic hypoglycemia with or without hyperammonemia, resemble those of FAODs, but normal acylcarnitine profile is present. Recessive mutations of HMG-CoA synthase (*HMGCS2*) and HMG-CoA lyase (*HMGCL*) deficiency are responsible of ketogenesis defects (61–65). Urine organic acid profiles during decompensation are usually dominated by secondary products of fatty acid oxidation, with a characteristic 4-hydroxy-6-methyl-2-pyrone (4-HMP) in HMG-CoA synthase deficiency. However, molecular analysis is essential for diagnosis (63). Avoidance of fasting and a high carbohydrate intake need to be maintained to prevent decompensations (61). HMG-CoA lyase deficiency is a life-threatening metabolic intoxication with a presentation mimicking a Reye syndrome including recurrent vomiting, severe non-ketotic hypoglycemia, metabolic acidosis, hyperammonemia, hepatomegaly, seizures, and coma triggered by a catabolic state such as infections or low dietary intake. Generally, the clinical onset is within the first year of age. However, epilepsy, lethargy, hepatomegaly, anemia and eating difficulties have been reported in older children (64, 65). Urine organic acids analysis shows a typical profile including high levels of 3-Hydroxy-3-MethylGlutaric, 3-MethylGlutaric, 3-MethylGlutaconic and 3-HydroxyIsovaleric acids, and an acylcarnitine profile revealing a high level of 3-hydroxy-isovalerylcarnitine with a decreased free carnitine concentration (66). The treatment is based on a protein- and fat-restricted diet. L-carnitine supplementation is recommended. The long-term outcome in older children is characterized by neurological complications such as epilepsy, muscular hypotonia and tremor associated with white matter lesions in the brain MRI (65).

#### 3.2.3 Ketolysis Defects

Ketolysis defects involve ketone utilization in extrahepatic tissues. The hallmark of decompensation is severe ketoacidosis. Two disorders may also present with hypoglycemia.

Beta-ketothiolase deficiency is an IEM that affects isoleucine catabolism and ketone body metabolism. Patients manifest intermittent ketoacidotic crises and hypoglycemia under catabolic stresses. Most patients developed their first crises between the ages of 6 months and 3 years. Neurological outcome, such as particularly extrapyramidal signs can occur, even in patients without any apparent decompensation (67). A characteristic increase in urinary 2-methyl-3-hydroxybutyrate and tiglylglycine, and a raise of C4OH levels at acylcarnitine profile are typical metabolic biomarkers (68).

Monocarboxylase transporter 1 (MCT1) mediates transport of pyruvate, lactate and ketone bodies across cell membranes. Patients with heterozygous or homozygous inhibiting mutations in *SLC16A1* present with moderate or profound ketosis and sometimes hypoglycemia during fasting or infections, within the first years of life (69, 70). In some patients migraine, exercise intolerance, developmental delay, microcephaly and abnormal MRI of the brain have been reported (71).

### 3.3 Disorders of Aminoacid Metabolism

#### 3.3.1 Organic Acidemias and Maple Syrup Urine Disease

In organic acidemias (methylmalonic, propionic and isovaleric acidemia) and maple syrup urine disease, the enzymatic deficiency (methylmalonyl-CoA mutase, propionyl-CoA carboxylase, isovaleryl-CoA dehydrogenase, and the branched chain ketoacids dehydrogenase complex, respectively) is responsible of a cellular intoxication with energy deprivation causing poor feeding, vomiting, seizures, respiratory distress, metabolic acidosis, ketonuria, increased serum ammonia, lethargy, encephalopathy progressing to coma. Inhibition of gluconeogenesis can lead to hypoglycaemia. Urinary organic acids, acylcarnitines and plasma aminoacids are essential diagnostic biomarkers. The conditions are typically detected on metabolic newborn screening and are treated by a nutritional regimen with limited intake of intact proteins (which contain branched chain aminoacids), while providing adequate branched chain aminoacids-free exogenous proteins *via* medical food, with the aim to reduce catabolism, promote anabolism and preserve normal growth and development (72–74).

#### 3.3.2 Hepato-Renal Tyrosinemia

Hepatorenal Tyrosinaemia or Tyrosinaemia Type 1 (HT1) is an autosomal recessive IEM caused by deficiency of the enzyme fumarylacetocetase in the pathway of tyrosine catabolism. This leads to the accumulation of tyrosine and toxic upstream intermediates such as succinylacetone, visible in urinary organic acids. Without treatment, HT1 patients suffer from liver failure and/or renal tubular dysfunction, and hepatocellular carcinoma (HCC) is a common long-term complication. HI is relatively common in untreated HT1. Pharmacological treatment consists of nitisinone along with a protein-restricted diet supplemented with aminoacid-mixtures free of tyrosine (75).

#### 3.3.3 Adenosine Kinase Deficiency

Adenosine kinase deficiency is a rare recessive disorder of methionine and adenosine metabolism with a severe clinical phenotype comprising mainly neurological and hepatic impairment and dysmorphisms. There is phenotypic variability from neurological to a multi-organ involvement, with hepatic steatosis to severe neonatal liver dysfunction, hypotonia, developmental delay and dysmorphisms. Many patients manifest epilepsy and HI, not always responsive to diazoxide. Biochemical markers are intermittent hypermethioninemia, increased plasma S-adenosylmethionine (SAM), S-adenosylhomocysteine (SAH) and adenosine, intermittent dicarboxylic aciduria with normal acylcarnitines. A methionine restricted diet improved clinical and biochemical signs in some patients (76).

### 3.4 Mitochondrial DNA Depletion Syndrome

Defects in any protein involved in mtDNA maintenance causing quantitative and qualitative defects are classified as mitochondrial DNA depletion syndrome (MDDS). Three clinical phenotypes have been described: hepatocerebral, encephalomyopathic and myopathic.

Mutations in deoxyguanosine kinase (*DGUOK*), mitochondrial inner membrane protein *MPV17*, polymerase catalytic subunit (*POLG*), succinate-CoA ligase GDP/ADP-forming subunit alpha (*SUCLG1*), twinkle MtDNA helicase (*TWNK*) and transcription factors A (*TFAM*) have been associated with hepatocerebral MDDS, with acute liver failure in infancy and hypoglycemia. *DGUOK* deficiency is one of the most frequent causes of hepatocerebral dysfunction (77). Recessive mutations of *POLG*, the main gene of mtDNA replication, are associated with a phenotype ranging from infantile hepatopathic poliodystrophy (Alpers-Huttenlocher syndrome) to sensory-ataxia neuropathy with dysarthria and ophthalmoplegia (SANDO), and spinocerebellar ataxia-epilepsy syndrome (SCAE) (78, 79). The Alpers-Huttenlocher syndrome is characterized by hepatic involvement with jaundice, cholestasis, hepatomegaly, elevated transaminases, evolving into liver failure with hypoglycemia associated with progressive neurologic symptoms and refractory epilepsy (79). Patients diagnosed with *DGUOK* mutations present with low birth weight and in a few weeks manifest neurological signs of rotatory nystagmus, hypotonia, and developmental delay, associated with hypoglycemia, raise of lactate and plasma alanine (80). *SUCLG1* deficiency has a characteristic methylmalonic aciduria at urinary organic acids (81).

## 4 Role of Genetics

In hypoglycemia-associated IEM, a rapid diagnosis is essential to establish appropriate and specific dietetic and pharmacological therapies, which are crucial for the short and long-term prognosis.

Although clinical characteristics of patients and laboratory signs may address the diagnosis, there are often overlapping phenotypes that induce uncertainty as well as mutations in multiple candidate genes can give rise to the same phenotype in the field of a specific disorder (e.g. HI, GSDs, MDDS). For these reasons, Sanger sequencing for single genes at a time is not applicable because time- and cost consuming (1, 82). In these conditions in which a genetic diagnosis allows to settle the more appropriate treatment, a rapid turnaround time is particularly significant. Genetic analysis influences decision making even in acute inpatient setting, as the case of HI in which the decision to candidate the patient to partial pancreatectomy depends on the finding of a recessive paternally inherited mutation in *ABCC8* or *KCNJ11*, which is suggestive of a focal form (13, 15). Furthermore, patients with KH without metabolic and hormonal biomarkers are often classified within idiopatic ketotic hypoglycemia (IKH). IKH has been mostly considered as a non-pathological condition of children with a fasting tolerance at the lower tail of the Gaussian normal distribution until school age (83). However, some children continue to present IKH until adulthood. Pathological KH has been defined as recurrent episodes with blood glucose < 70 mg/dl (3.9 mmol/L) and betahydroxybutyrate ≥ 1.0 mmol/L, without any trigger of fasting or infections. Pathological KH may be due to underlying diseases, e.g. GSDs, defective MCT and genetic syndromes or to novel diseases that can be identified by whole exome sequencing (WES). The treatment consists in prevention of hypoglycemia and protein deficiency by adequate supplementation of carbohydrates and proteins, with uncooked cornstarch, or continuous tube feeding by night. Failure to settle a proper diagnosis of IKH may lead to undertreatment (71). Indeed, the ketotic forms of GSDs are underrecognized because endocrinological and metabolic parameters are unremarkable during investigations for hypoglycemia (84). Brown et al. found variants in genes causing GSDs (including type 0, VI, IXa, IXb, IXc) in 12% of IKH patients without hepatomegaly, by performing Sanger sequencing on five genes (*GYS2*, *PYGL*, *PHKA2*, *PHKB*, and *PHKG2*) (85). This finding was unexpected, because GSDs are usually suspected in case of hepatomegaly and raise of transaminases. This finding led other physicians to perform NGS in a patient with frequent IKH without hepatomegaly or elevated liver enzyme levels, unravelling a rare variant in the *PHKA2* gene. In those cases, KH patients should be treated with hyperproteic diet, similarly to GSD type IXa (86).

By use of trio exome sequencing in patients with IKH, researchs have identified variants also in *SLC16A1* (MCT1), *NCOR1*, *IGF2BP1*, *SGLT2* and *NEK11* as potential novel causes of unexplained KH (69, 87, 88).

In the field of GSDs, Kim at al. reported the gene panel for GSDs as a useful tool to confirm the diagnosis of GSD IX subtypes. They clarified genotype, phenotype and long-term outcomes of patients with GSD type IX. Furthermore, they reported the development of hepatocellular carcinoma in a patient with GSD type IXc (42).

Seven new GSD type 0 patients with variable phenotypes were found by a gene panel in a recent report. Seven variants were novel, and four were classified as of uncertain clinical significance (VUS). Their frequency in the healthy cohort was extremely low, but there were not enough supporting criteria for interpreting these variants as pathogenic or likely pathogenic. Predictive systems gave different interpretations. All patients showed hyperglycemia and hyperlactatemia three hours after feeding, with some having ketones in blood and urine, others only in blood. One patient showed enlarged liver (89). In another study, two patients lacking of postprandial hyperglycemia/hyperlactatemia were diagnosed with GSD typo 0 by targeted NGS (1). Another report described a diagnosis of GSD type 0 through WES in a patient with a nonclassic presentation (90). In other two studies exome sequencing was used in pediatric and adult patients with GSDs affecting both liver and/or muscle. The former reported a presumptive diagnostic yield of 65% by targeted exome sequencing (91). In the other, the diagnostic yield was 43% with clinical exome sequencing and 25% with targeted exome sequencing (92).

In the Ponzi study, the use of a gene panel for hypoglycemia showed a mutation detection rate of 59% in GSDs and other carbohydrate metabolism disorder subgroup. A diagnosis of GSD type IXa was established in a patient with hemizygous deletion in *PHKA2* gene. GDS type III was diagnosed in a patient with homozygous deletion in *AGL* gene. Another child was diagnosed with GSD type IXc, carrying an unreported biallelic missense mutation in *PHKG2* gene which strongly correlated with the observed phenotype. Unexpectedly, two GSD type 0, one HI and one HFI were diagnosed from the no candidate gene class. These three patients presented with unusual findings: variable fasting tolerance, intermittent ketonemia and absence of postprandial hyperglycemia/hyperlactatemia in GSD type 0; hypoketotic hypoglycemia responsive to glucagon, with increased NEFA in HI; absence of fructose containing foods aversion, fasting hypoketotic hypoglycemia responsive to glucagon, suppressed NEFA, increased liver enzymes and renal tubular dysfunction in HFI (1).

Fructose-1,6-phosphatase deficiency is often misdiagnosed. A study described four patients with recurrent hypoglycemia diagnosed *via* targeted NGS panel. In three of them, the onset of hypoglycemia was in the first two years of age. However, without a clear diagnosis, the families were not aware of how to prevent the attacks, thus they experienced several life threatening events until the genetic diagnosis was settled (93). In a recent case report, the trio exome sequencing revealed the diagnosis postmortem (“molecular autopsy”) of cytosolic PEPCK deficiency in a 3-year-old boy with an initial suspicion of a febrile seizure during infection, evolved rapidly in hypoglycemia and cerebral edema. The metabolic screening showed elevated urinary lactate and Krebs cycle intermediates, indicating an energy deficiency. The postmortem diagnosis had an important psychosocial impact for the whole family and provided the chance of a predictive testing to all family members at risk (94). Unexplained coma and sudden death in an apparent healthy infant is a dramatic family life event. Several studies reported on high rates of emotional distress in parents of children with undiagnosed conditions (95).

Rojnueangnit et al. described two unrelated infants with atypical presentation which expanded the phenotype of *HMGCS2* deficiency. During acute episodes, steatorrhea and dyslipidemia (increased triglycerides, VLDL, and LDL, along with decreased HDL) occurred, both previously unreported. Both patients manifested encephalopathy, hypophosphatemia, hyperphosphaturia and proteinuria. One patient presented metabolic acidosis without hypoglycemia. The urinary 4-HMP was not detected. Trio WES revealed compound heterozygous for *HMGCS2*. Hypoglycemia with impaired ketogenesis may have determined an increased lipolysis with raise of NEFA along with triglycerides (96). Although the presence of 4-HMP in urine has been reported as a biomarker of *HMGCS2* deficiency (63), the substance is not always present (97, 98) and likely only appears during decompensation (63). Furthermore, the authors showed that metabolic acidosis without hypoglycemia can be a metabolic feature of *HMGCS2* deficiency (96). Elsewhere, the patient with a ketogenesis defect showed homozygosity for a not reported variant in *HMGCS2* captured by gene panel, predicted as pathogenic *in silico*. A revaluation of his urinary organic acid profile confirmed the presence of the characteristic pattern (1).

In the Ponzi study, the 78% of patients with a single candidate gene, 49% of patients with multiple candidate genes, and 33% with no candidate gene reached a diagnosis. The diagnostic yield of the gene panel was 48% for HI, 59% for GSDs and other carbohydrate disorders, 66% per FAODs and ketogenesis defects, and 67% for mitochondrial disorders (1).

## 5 Role of Genetics in Findings Novel Genes

WES has been frequently used to map novel genes involved in the pathogenesis of unexplained hypoglycemia. Variants in *SLC16A1* (MCT1), *NCOR1*, *IGF2BP1*, *SGLT2* and *NEK11* have been identified as potential novel causes of unexplained KH (69, 87, 88).

MCT deficiency was described above: inhibiting mutations are responsible for moderate or profound ketosis and sometimes hypoglycemia (69, 70). The other genes are involved in various processes that might affect gluconeogenesis, glycogenolysis and translational regulation.

The NCOR1/HDAC3 complex is involved in the regulation of liver phosphoenolpyruvate carboxykinase (PEPCK), glucose 6-phosphatase catalytic (G6PC) and hepcidin. The KH patient with an *NCOR1* mutation had iron deficiency anemia as additional feature, likely due to hepcidin overexpression (87). Remarkably, patients with GSD type Ia (*G6PC* mutations) may present also iron deficiency anemia caused by increased secretion of hepcidin from hepatic adenomas (30).

IGF2BP1 regulates the activity of several proteins, including IGF2 that is involved in cell growth and differentiation and activates the insulin receptor. *In vitro*, IGF2BP1 suppresses the translation of IGF2 mRNA. Therefore, increased IGF2 levels will be caused by loss of function mutations of *IGFBP2*. Remarkably, impaired glycogenolysis and gluconeogenesis with consequent hypoglycemia and suppressed insulin secretion have been found in patients with IGF2-producing tumors.

Dominant mutations in *SLC5A2*, which encodes the sodium glucose co-transporter 2 (SGLT2), lead to familial renal glucosuria (FRG). The KH patient with a *SLC5A2* mutation presented hypoglycemia with intermittent glucosuria, suggesting that FRG may cause KH in infancy.

The mitosis gene A-related kinase 11 gene *NEK11* has not been linked to other diseases in humans than KH. Hypoglycemia was reported in a *NEK11*- mouse model. The described KH patient with a heterozygous *NEK11* mutation showed glucagon unresponsive hypoglycemia, migraine, cognitive disability, motor impairments, mild hepatopathy, and decreased plasma IGFBP3 (71, 87).

Recently, the use of exome sequencing allowed the detection of a single *CACNA1D* (encoding for the L-type voltage-gated calcium channel) activating mutation in a syndromic child with neurodevelopmental delay, aortic insufficiency and HI requiring diazoxide therapy. As pancreatic L-type voltage-gated calcium channels are involved in insulin secretion, mutations in *CACNA1D* may have a potential pathogenic role (99). A previous patient with primary aldosteronism and without HI had been described (100). A second patient with developmental delay, hypotonia, aortic insufficiency, primary hyperaldosteronism, and facial dysmorphisms was recently diagnosed by clinical exome sequencing, identifying a novel *de novo CACNA1D* missense mutation, thus confirming the implication of *CACNA1D* for primary aldosteronism and HI. The genetic diagnosis led to add nifedipine to the therapy that was effective for glycemic and pressure control and muscle tone (99, 101). Nifedipine readily permeates the blood-brain barrier and thus may also inhibit L-type voltage-gated calcium channels in the brain. Indeed, the start of nifedipine was associated with an improvement of hypotonia (101).

A further gene recently likely associated with HI is the developmental transcription factor, forkhead box A2, *FOXA2*, in which a *de novo* heterozygous mutation was found by WES in a child with HI, hypopituitarism, liver, lung and gastrointestinal malformations, choroidal coloboma and dysmorphisms (102). Foxa2 is an important developmental transcription factor for the formation of midline structures and endoderm derived organs including the pancreas, and regulates insulin secretion from pancreatic β-cells. The mechanisms are poorly understood. However, a *FOXA2* mutation might alter the expression of SUR1 and/or Kir6.2, or activate the transcription of *HADH* that encodes L-3-Hydroxyacyl-CoA-dehydrogenase (HADH), an enzyme involved in the penultimate step of the β-oxidation pathway. Furthermore, it could increase the GLUT2 expression in pancreatic β-cells, promoting the glucose transport through the cell membranes and thus enhancing the insulin secretion. Foxa2 could also play a role in the development of the pancreas, through the regulation of *Pdx1*, a homeobox gene essential for pancreatic development. Finally, Foxa2 has also been implicated in the regulation of Hnf4a and Hnf1a, involved in HI and monogenic diabetes (102).

Recent description of loss of function mutations in *EIF2S3*, discovered by exome sequencing of the X-chromosome, were also been associated to hypoglycemia, hypopituitarism and pancreatic dysfunction in three boys. The heterotrimeric GTP-binding protein eIF2 forms a ternary complex with methionyltRNA promoting the onset of protein synthesis. Mutations in *EIF2S3* (X-linked), encoding the eIF2γ subunit, have been associated with cardinal phenotypic features of microcephaly and intellectual disability, usually as part of MEHMO syndrome characterized by short stature, hypogonadism, epilepsy, significant intellectual disability and microcephaly. The three patients presented a novel milder phenotype, with mild learning difficulties, short stature, hypogonadism and glucose dysregulation with HI and post-prandial hyperglycaemia (as shown at prolonged glucose tolerance test with hypoglycemia at baseline and at 5 hours with a detectable insulin, and hyperglycemia at 2 hours). They were treated with rhGH, thyroxine, diazoxide and chlorothiazide. The early molecular diagnosis might have contributed to the prevention of severe neurodevelopmental delay, which could be related to untreated unrecognized hypoglycemia (103).

Recessive homozygous c.4910G>A mutations of *LRP4* have been recently discovered by clinical exome sequencing to be associated to unexplained KH in two siblings affected by Cenani-Lenz syndactyly, characterized by skeletal abnormalities, dysmorphisms, renal hypoplasia, deafness, congenital cataract (104). However, since LRP4 is a receptor involved in cell adhesion and receptor-ligand interactions in bone, kidney and nervous system, its putative role in the pathogenesis of hypoglycemia might be coincidental.

Recessive loss of function mutations of *DNAJC3* have been found by exome and genome sequencing to cause early HI evolving into diabetes for insulin insufficiency, growth retardation and neurodegeneration in four children (105, 106). Patients presented demyelinating neuropathy, learning difficulties, hypothyroidism, microcephaly, retinal dystrophy, sensorineural deafness. DNAJC3 (P58^IPK^) is member of the heat shock proteins produced in the endoplasmic reticulum to counter cell stress and having a protective role against neurodegeneration. The gene is expressed in endocrine cells such as pancreas and thyroid. Early onset HI might be due to a disturbance of the balance between β-cell apoptosis and proliferation. HI is responsive to diazoxide (105). Furthermore, P58^IPK^ null mice developed inhibition of C/ebpα which regulates gluconeogenesis and lipogenesis in the liver. These mice manifested fatty liver, hypoglycemia and depletion of hepatic glycogen (107).

## 6 Discussion and Closing Remarks

**Figure 1:**
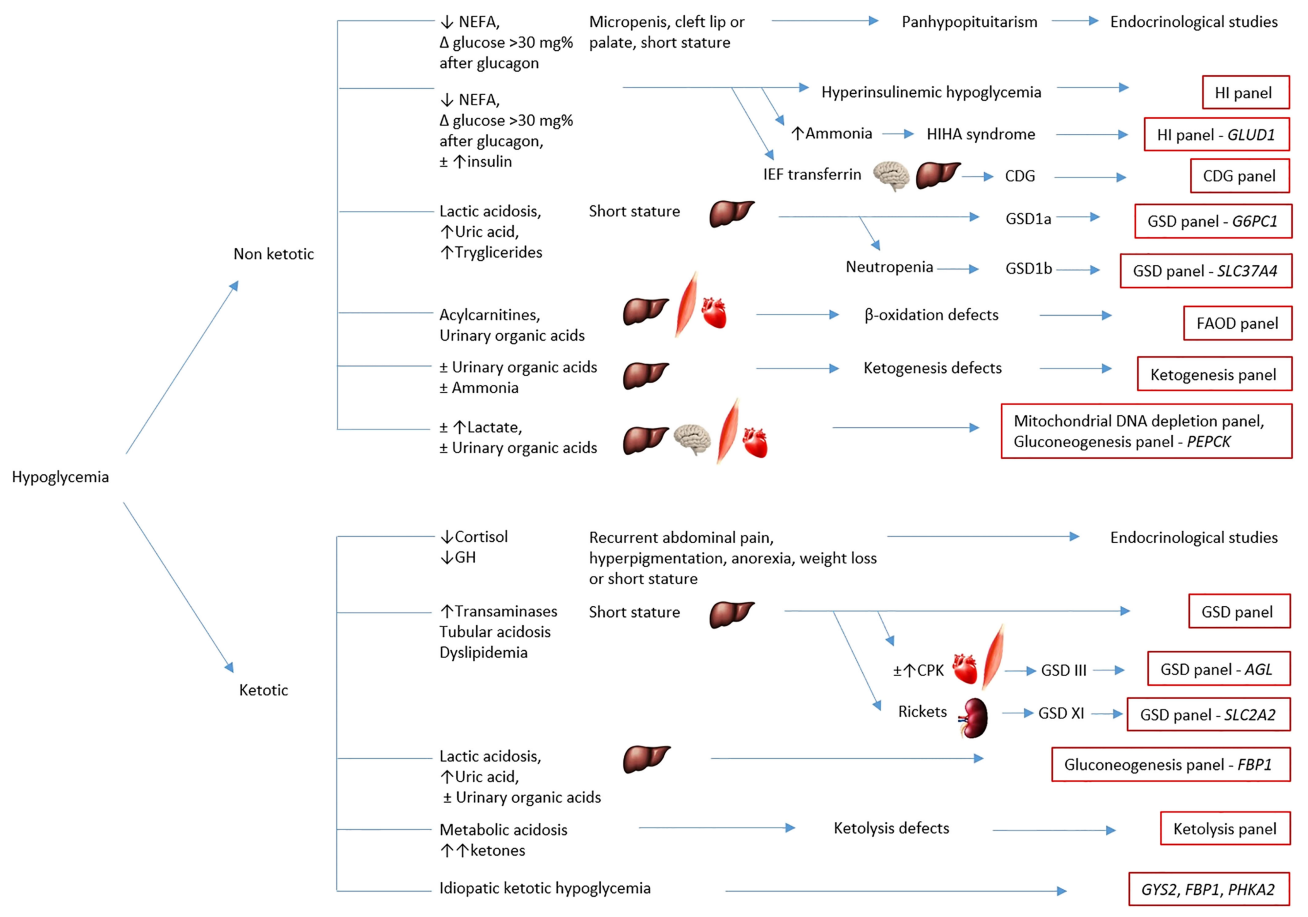
Simplified chart of the diagnostic algorithm for hypoglycemia. The chart summarizes the main causes of hypoglycemia and classifies them according to clinical and biochemical characterization for targeted metabolic gene panel testing. The eventual hepatic, cardiac, muscular or neurological involvement is outlined by the corresponding anatomical pictures. HI, hyperinsulinemic hypoglycemia; HIHA syndrome, hyperinsulinism-hyperammonemia syndrome; CDG, congenital disorders of glycosylation; GSD, glycogen storage disorders; FAOD, fatty acids oxidation defects; IEF transferrin, isoelectrofocusing of transferrin.

In the last years the molecular approach for sequencing genetic informations at scale has changed substantially. Historically, genetic analysis consisted on single gene testing at a time. Lately, the new NGS technology made it possible to carry out large molecular characterization of patients within an useful timeframe. This was particularly applicable for the diagnosis of IEM presenting with hypoglycemia, because of the genetic heterogeneity of these conditions (1) (**Figure 1**, **Table 1**).

Targeted gene panels capture variants within a few target genes (10s to 100s of genes) and are commonly used for diagnostic purposes, as they generate manageable quantities of data with a low cost and turnaround time. The list of all genes is pre-established and needs to be updated along with new disease genes discovering (**Table 1**). A further approach is the use of virtual panels to make available a clinical exome (Mendelioma, for sequencing all genes associated to Mendelian inheritance) data set that can be reviewed overtime at no additional cost to test other candidate genes (108, 109). However, in instances of diagnostic uncertainty, WES has a higher diagnostic yield. WES detects all variants within the entire protein-coding region (∼20,000 genes), representing less than 2% of the genome but containing the ∼ 85% of known disease-causing variants. WES is suitable for novel gene discovery in idiopathic conditions. However, non-coding and structural variants cannot be captured, and the gene coverage may be variable. Therefore, the detection of mutations in deep intronic regions may be missed by the use of gene panels or WES, or if a detected mutation was considered as a nonpathogenic variant. Whole-genome sequencing (WGS) captures variants from the entire genome. Besides revealing the ∼98% of the non-coding regions, it provides a better coverage and analysis of the coding regions. Indeed, WGS revealed large deletions in genes associated to some IEM, not detected by WES. Nevertheless, reliable tools to interpret non-coding variants are still not available, and cost and turnaround time are high (110, 111). However, the buildup of WES and WGS data in the human population and the systematic use of trio sequencing will possibly increase the diagnostic yield in unexplained conditions (79). Though, as WES and WGS lean on short-read sequencing, there are genomic regions still difficult to sequence such as tandem-repeat expansion, large deletions and insertions, and complex chromosomal structural abnormalities. In those cases, long-read sequencing technologies could unveil such rare variations. Recent studies about the application of long-read sequencing to pathogenic variants in rare diseases not detected by conventional NGS techniques gave promising results (112).

A wide proportion of existing variants are classified as VUS, for which functional studies or computational tools are necessary to clarify the pathogenicity. It has been estimated that the probability to detect a VUS is as higher as larger is the number of genes tested: 36% in multigene panels and 73% in exome sequencing (113). To establish a proven genetic diagnosis, a disease causing variant should be detectable and clinically interpretable. Various computational prediction systems have been developed to interpret the impact of those variants on clinical phenotypes (110). However, international guidelines are insufficient to unravel the pathogenicity of certain findings (110, 114). As more information become gradually available, VUS may be redefined as pathogenic/likely pathogenic or benign/likely benign (113). In communicating genetic VUS, there is a risk of overdiagnosis and overtreatment if they were inappropriately classified as pathogenic. Multidisciplinary cooperation is prominent for interpreting the significance of genomic results (111).

**Figure 2:**
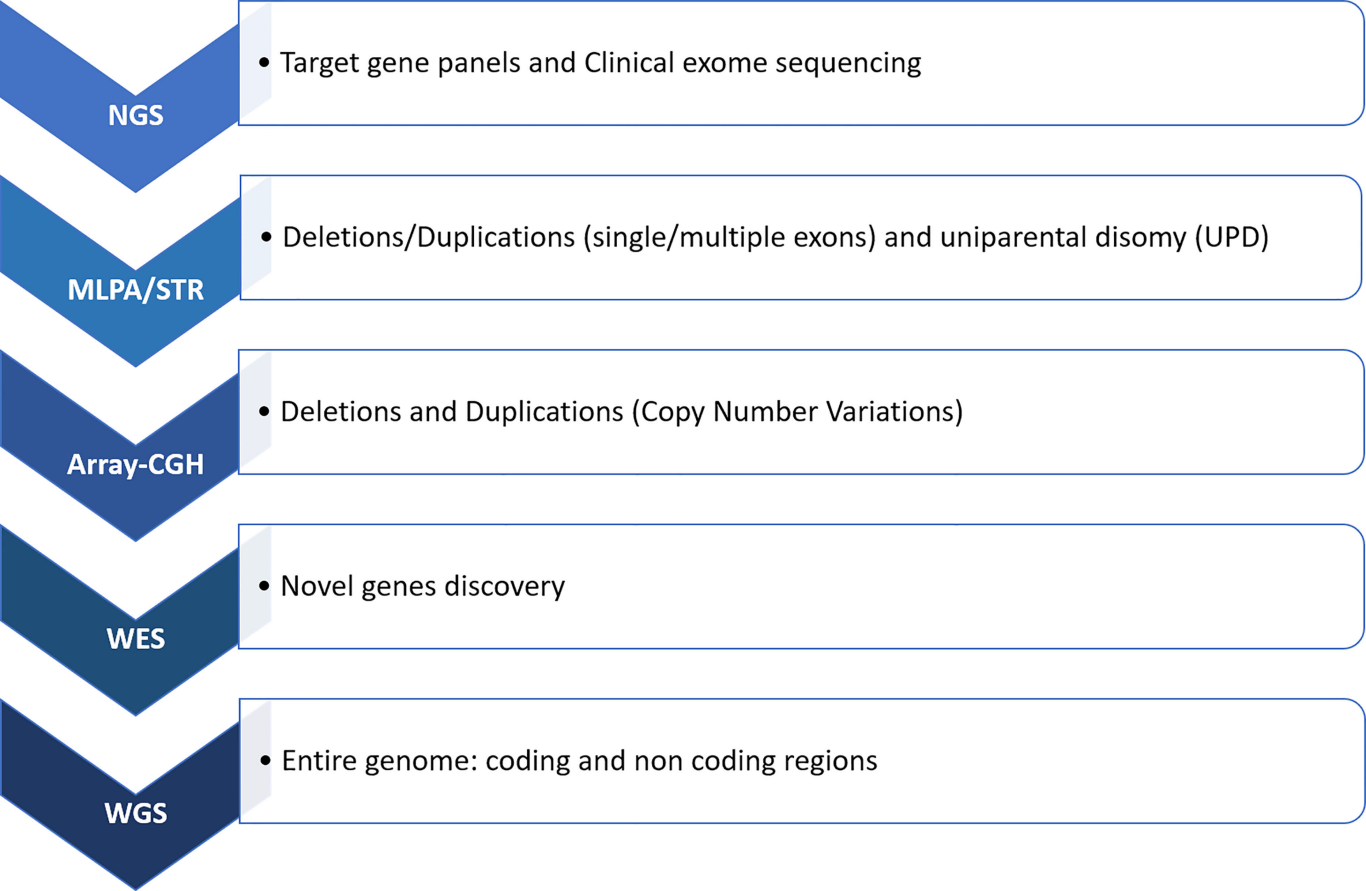
Molecular workflow for hypoglycemic conditions. In cases of not conclusive results from NGS, a research for deletion/duplications should be performed through MLPA and array-CGH. Integration with STR can be worthwhile when an uniparental disomy is suspected. Subsequently, WES and WGS techniques can be used for sequencing the entire genetic code. NGS, next generation sequencing; MLPA, multiple ligation-dependent probe amplification; array-CGH, comparative genomic hybridization array; STR, short tandem repeats; WES, whole exome sequencing; WGS, whole genome sequencing.

In cases of negative or partial NGS results of targeted gene panels and clinical exome sequencing, but strong evidence of biochemical or clinical phenotype or discrepancy with segregation studies, other techniques should be used, including multiple ligation-dependent probe amplification (MLPA) and high-resolution comparative genomic hybridization (CGH) array (1) (**Figure 2**). In a recent case of GSD type III, the use of single nucleotide polymorphism (SNP) array and short tandem repeats (STR) segregation study revealed for the first time a paternal isodisomy of chromosome 1 (115). Conversely, NGS approach can help to make a diagnosis in case of negative biochemical results as in some GSDs, or in *HMGCS2* mutations in which the characteristic biomarker is not always present (96–98).

The importance to make a rapid differential diagnosis lies in establishing the appropriate therapeutic approach, such as specific nutritional intervention regarding GSDs and FAODs type and GCK-HI, or medical therapy in case of GSD type Ib as well as *HNF4a*/*SLC2A2* gene mutations, which share a similar phenotype but different treatment, or surgical strategies in case of focal-HI.

Identification of new rare disease genes may influence the impact of receiving a diagnosis, because often the long-term effects of new genetic conditions are unknown. The agnostic approach of WES and WGS is also challenging our previous knowledges of existing genetic diagnoses, when pathogenic variants give raise to unexpected clinical pictures. Therefore, this approach is allowing to expand the phenotypic characterization of rare diseases (111), such as the above reported cases of GSD type IXa (85), *HMGCS2* deficiency (96), MEHMO syndrome (103).

By using the NGS approach in IEM presenting with hypoglycemia, Ponzi et al. demonstrated that it provided a rapid diagnosis in 45% of patients in whom clinical and laboratory findings did not allow to identify a single candidate gene. Furthermore, invasive or expensive procedures have been avoided, such as liver biopsy for suspected disorders of carbohydrate metabolism. However, NGS will not replace the metabolic work-up, which is critical to drive the molecular analysis toward those clusters of genes involved in specific pathways (**Figure 1**). In addition, the biochemical and clinical phenotype addresses data interpretation regarding the finding of possible disease-causing variants at first reported as VUS, or the discovery of new disease genes (1). Remarkably, in a huge retrospective study of WES applied as a primary newborn screening test for 48 IEM in an 8.5-year population scale cohort in California (the NBSeq project) an 88% overall sensitivity and 98.4% specificity was estimated. Conversely, the current screening performed with MS/MS analite-based shows 99.0% sensitivity and 99.8% specificity in the same cohort (110). Furthermore, a synergistic association with other biotechnologies, such as enzymatic assay for residual activity in FAODs, may allow a better characterization of new variants in order to define pathogenicity, customize follow-up and avoid overtreatment (116). Finally, the NGS approach allows genetic counseling for recurrence risk in further pregnancies (1), prenatal and preimplantation diagnosis (43).

In conclusion, innovative diagnostic protocols are required for genetically heterogeneous disorders. NGS can routinely be easily used as a standard diagnostic tool with a straightforward workflow to identify disease-causing mutations. It allows an early detection of mutations, with a high standard in terms of coverage and quality, is cost-effective and has a rapid turnaround time. These data promote the expanding application of the NGS technologies for IEM presenting with hypoglycemia, because of their genetic heterogeneity and complex phenotypes, variable or atypical presentations, for a more appropriate clinical management and genetic counselling (79). Multidisciplinary input and collaboration are increasingly key for addressing the analysis and interpreting the significance of the genetic results, allowing rapidly their translation from bench to bedside.

## Author Contributions

AM conceptualized and designed the study, searched for literature, drafted the initial manuscript, reviewed and approved the final manuscript as submitted. FL, AN, and CD-V wrote sections of the manuscript, critically reviewed the manuscript and approved the final manuscript as submitted. All authors contributed to manuscript revision, read, and approved the submitted version.

## Conflict of Interest

The authors declare that the research was conducted in the absence of any commercial or financial relationships that could be construed as a potential conflict of interest.

## Publisher’s Note

All claims expressed in this article are solely those of the authors and do not necessarily represent those of their affiliated organizations, or those of the publisher, the editors and the reviewers. Any product that may be evaluated in this article, or claim that may be made by its manufacturer, is not guaranteed or endorsed by the publisher.
